# Variation in Risk of COVID-19 Infection and Predictors of Social Determinants of Health in Miami–Dade County, Florida

**DOI:** 10.5888/pcd17.200358

**Published:** 2020-10-08

**Authors:** Imelda K. Moise

**Affiliations:** 1 Department of Geography and Regional Studies, University of Miami, Coral Gables, Florida

## Abstract

Miami–Dade County zip code–level (N = 91 zip codes) coronavirus disease 2019 (COVID-19) cases (N = 89,556 as of July 21, 2020) reported from the Florida Department of Health were used to estimate rates of COVID-19 per 1,000 population at the census block group level (N = 1,594 study block groups). To identify associations between rates of COVID-19 infections and multidimensional indexes of social determinants of health (SDOH) across Miami–Dade County, Florida, I applied a global model (ordinary least squares) and a local regression model (geographically weighted regression). Findings indicated that a social disadvantage index positively affected COVID-19 infection rates, whereas a socioeconomic status and opportunity index and a convergence of vulnerability index had an inverse but significant connection to COVID-19 infection rates over the study area. Rates of COVID-19 infections were localized to specific geographic areas and ranged from 0 to 60.75 per 1,000 population per square mile.

**Summary**

**What is already known about this subject?**

Coronavirus disease 2019 (COVID-19) continues to have a disproportionate impact on certain populations in the United States, particularly racial and ethnic minorities and people with underlying medical conditions.

**What is added by this report?**

By August 19, 2020, Miami–Dade County accounted for 25% of all new COVID-19 cases reported in Florida. The positive association between a social disadvantage index and COVID-19 rates reflects the localized social networks and neighborhood social disadvantage. In addition, in Miami–Dade County, Florida, COVID-19 is localized to specific geographic areas.

**What are the implications for public health practice?**

COVID-19 infections are associated with socioeconomically vulnerable groups or areas, indicating a need for place-based and policy-level strategies or social protection policies that protect vulnerable social groups (eg, children, older adults, and single parent households).

## Objective

By August 19, 2020, Miami–Dade County accounted for 25% (148,093) of all new coronavirus disease 2019 (COVID-19) cases (N = 584,047) reported in Florida (1). Of particular concern is COVID-19’s effect on vulnerable populations, such as minorities and people with chronic disease, and its linkage to social determinants of health (SDOH) (2,3). According to Healthy People 2030, SDOH (poverty, unequal access to health care, lack of education, and social conditions) affects a wide range of health, functioning, and quality of life outcomes (4). The SDOH also exacerbate health outcomes for vulnerable populations (5–7). The Centers for Diseases Control and Prevention reported that almost all people (94%) who died of COVID-19 in the United States had at least 1 underlying medical condition (8). A recent study also found overlaps in rates of COVID-19 infection and chronic disease (9). Therefore, finding effective ways to recognize the features that influence disadvantaged populations during a pandemic and to intervene is a persistent problem facing public health. The objective of this study is to quantify different SDOH indexes, examine the measures of these indexes on rates of COVID-19 infections, and determine the spatial variation in COVID-19 risk across census block groups in Miami–Dade County, Florida.

## Methods

Confirmed data on the number of COVID-19 cases at the zip code level (N = 91 Miami–Dade County zip codes) as of July 21, 2020, were obtained from the Florida Department of Health COVID-19 Data and Surveillance Dashboard (1). COVID-19 data are reported only at large geographic levels (city, zip code, or county), which can mask small area variations (10) such as those occurring at the census block group level where improvements in health outcomes are most needed. Therefore, I used areal interpolation, a kriging-based disaggregation technique. A major advantage of areal interpolation is that it estimates data across different spatial aggregation units (eg, zip codes) and across units missing data (eg, census block groups) to produce a smoothed surface map of COVID-19 infection rates (11). I used the following parameters: a spherical model, a lag distance of 1,000 meters, and I limited the number of block groups in the prediction to 4 neighbors. The predicted data fit best when the model type was K-Bessel and the number of lags was 12, and all other inputs were set to default. Rates of COVID-19 infections per zip code per 1,000 population were calculated before areal interpolation.

Census block group level indicators were obtained from the US American Community Survey (ACS) 5-year estimates (2014–2018) (12). The 15 measures of social and neighborhood factors commonly reported as influencing health outcomes and common to several SDOH frameworks (12) were reduced to 3 indexes by using a principal components analysis (PCA) interpreted as signs of socioeconomic status and opportunity index (SESOI), social disadvantage index (SDI), and convergence of vulnerability index (CVI) (Table). The benefit of PCA is that it produces a new set of uncorrelated measures as a linear grouping of the initial measures and describes as much of the initial variation as possible. Contrary to a similar index construction study (13), this study’s results were not consistent with a hypothesis of equal significance of measures in the indexes (eg, predefined measure set). Such measures, for example, did not adequately represent SES for the study area.

**Table:** Component Loadings for the 15 Census Block Group Measures Included in 3 SDOH Components, Miami–Dade County, Florida, 2020

Measure	SES and SESOI	Social Disadvantage Index	Convergence of Vulnerability Index
Component variance^a^	41.78	12.2	10.7
No vehicle	.839	—	—
Renter	.803	—	—
Rent burden	.793	—	—
Limited English proficiency^b^	.679	.570	—
Median household income	−.675	—	—
Living in poverty	.586	—	.544
People with disabilities	.478	—	.436
Crowding	—	—	—
Single parent–headed households	—	.885	—
Households with children aged <18 y	—	.742	.464
Households with one or more people aged ≥65	—	.725	—
No high school diploma	—	.628	—
Uninsured people	.439	.529	.518
Race/ethnicity (all people except non-Hispanic White)	—	—	.862
Unemployed, aged ≥16	—	—	.720

Abbreviation: SDOH, social determinants of health; SES, socioeconomic status; SESOI, socioeconomic status opportunity index; —, excluded low values (below 0.30).

a Values are percentage variance. Extraction method: principal component analysis. Rotation method: Varimax with Kaiser Normalization (rotation converged in 6 iterations). Data source: US Census American Community Survey 5-year Estimates (2014–2018) (12). The 3 components reflect the convergence of predisposing, enabling, and need attributes of COVID-19 infection risk across census block groups in Miami–Dade County. SES and Opportunity Index include socioeconomic measures of poverty, income, person with limited English proficiency, and physical measures of housing characteristics (eg, renters, rent burden, and crowding) including vehicle access that have been linked to distinct health behaviors and outcomes. The Social Disadvantage Index includes demographic measures of socioeconomically vulnerable groups or areas with a high percentage of people with limited English proficiency, single parent households, households with children aged younger than 18 years, older adults (aged ≥65 y), people with less than a high school education, and uninsured people, which reflect localized social networks and neighborhood social disadvantage. The Convergence of Vulnerability Index includes measures of service environment or areas with a high proportion of people living in poverty, people with disabilities, children aged younger than 18 years, uninsured people, people with minority status, and unemployed people aged 16 or older. These measures compound already poor health profiles of vulnerable groups, increasing their risk of morbidity and mortality from COVID-19.

b Limited English proficiency crossed the SES and Opportunity Index and the Social Disadvantage Index. Living in poverty and people with disability crossed the SES and Opportunity Index and Convergence of Vulnerability Index. Households with children aged 18 years or younger crossed both the Social Disadvantage Index and the Convergence of Vulnerability Index. Uninsured people crossed all indexes.

**Figure 1:**
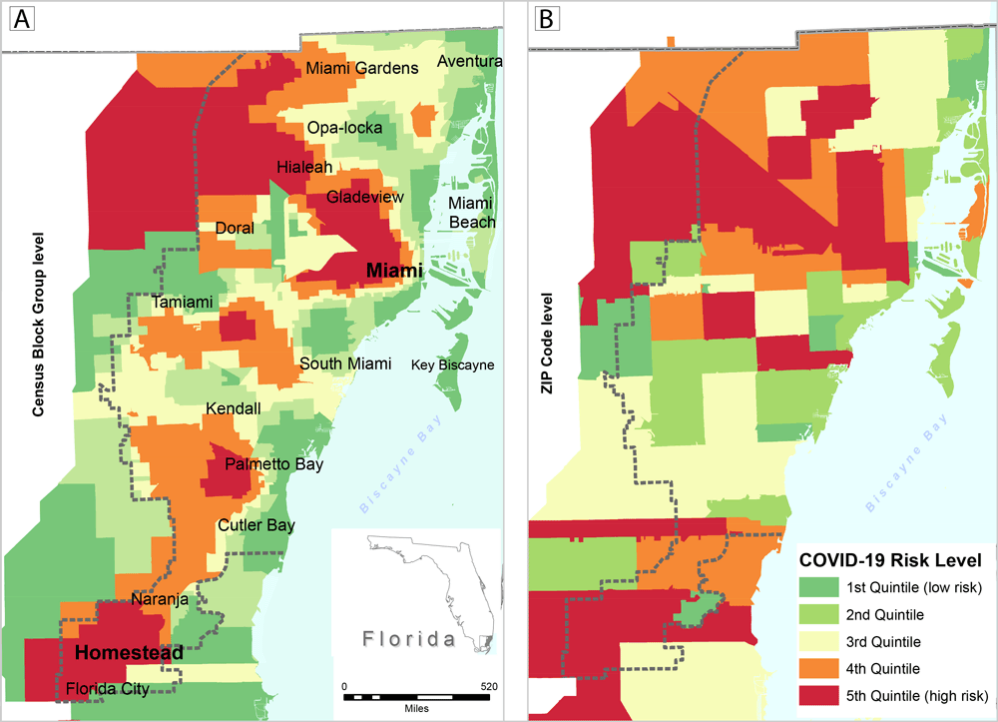
Map A shows estimated census block group level COVID-19 rates per 1,000 population for Miami–Dade County, Florida (generated with areal interpolation) based on zip code level rates. Map B is the same map as A but at a larger geographic area of zip codes. Data are for the 89,556 confirmed cases of COVID-19 reported as of July 21, 2020, in the Florida Department of Health COVID-19 Data and Surveillance Dashboard. Maps show rates (by quintiles) per 1,000 population.

**Figure 2:**
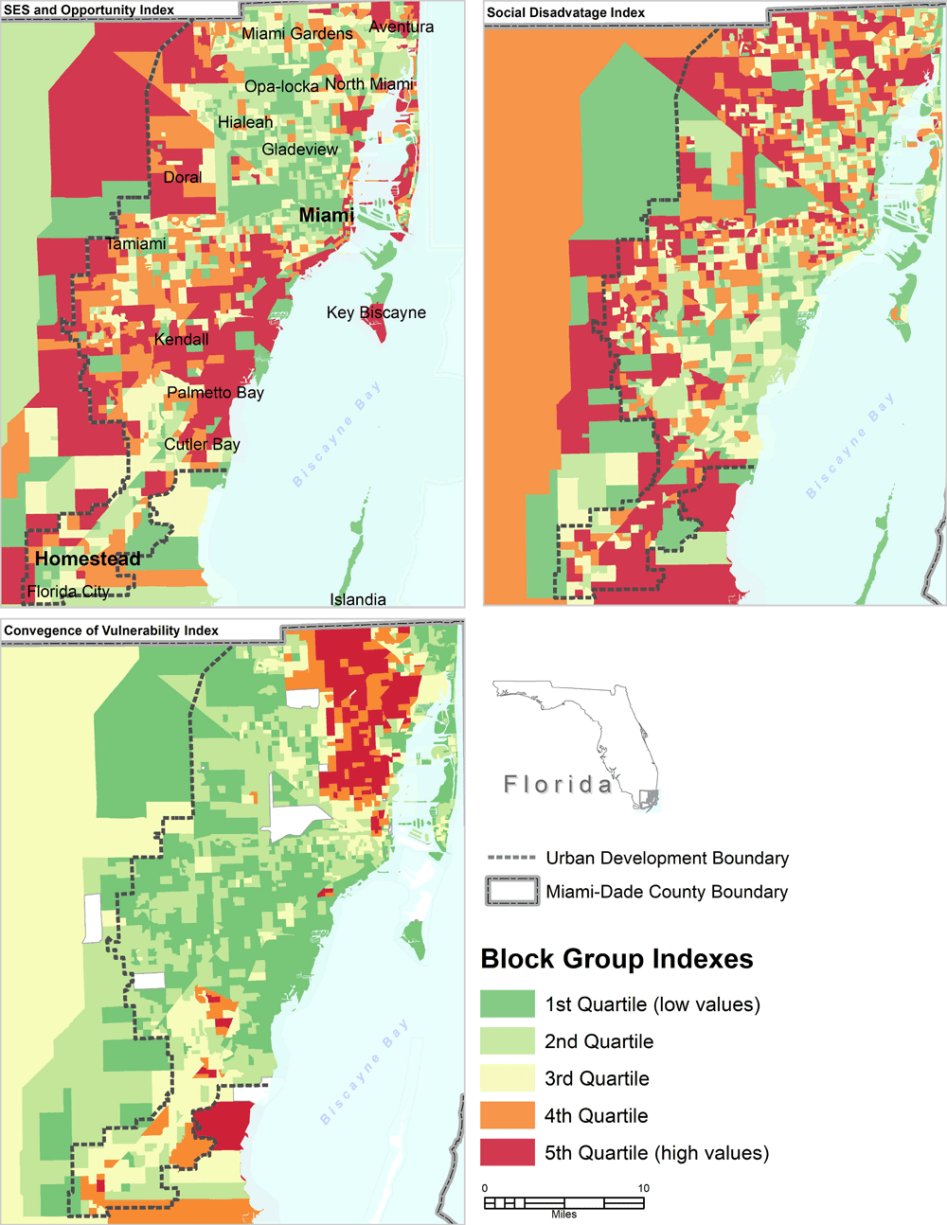
Maps of selected composite measures of 3 social determinants of health indexes for census block groups in Miami–Dade County, Florida: socioeconomic status and opportunity index, social disadvantage index, and convergence of vulnerability index. Abbreviation: SES, socioeconomic status.

The eigenvalue for the SESOI was 6.266, and it explained 41.8% of the variance. The eigenvalue for the SDI was 1.83, and for the CVI was 1.61. The SDI and CVI indexes explained 12.2% and 10.7% of the variance, respectively. To determine the dominant measures in each principal component, the cutoff measure loading of 0.30 for the component was used, which is common practice in the literature. Quintiles maps were generated by using the ArcGIS software version 10.5 (Esri) to visualize census block group level COVID-19 infection rates compared with zip code–level rates (Figure 1) and composite measures (Figure 2). I used ordinary least squares (OLS) for global regression rather than geographically weighted regression (GWR) by using the MGWR version 2.2 software (Microsoft Corp) to identify associations between rates of COVID-19 infections and the SDOH multidimensional indexes across Miami–Dade County, Florida. The model was set as COVID-19 rates = β_0_ + β_1_ SESOI + β_2_ SDI + β_23_ CVI + ɛ. β_0_ and β_1_ were the regression coefficients and ɛ was the model random error. The Akaike information criterion (AIC) was used to assess goodness of fit between the 2 models.

## Results

A total of 89,556 confirmed COVID-19 cases were reported in Miami–Dade County during the study period. The social disadvantage index was a better predictor of COVID-19 infections (*F*_2, 1,584_ = 75.83; *P* < .001) compared to the SESOI or the CVI, which suggests a need for policy-level strategies or social protection systems for vulnerable social groups (eg, children, older adults, single parent households). When comparing the OLS model with GWR AIC values, the AIC values show that both models perform roughly the same (GWR, 4,326.972 vs OLS, 4,327.199; adjusted *R* ^2^, 0.120 vs 0.122), with the GWR model being slightly favored. Therefore, reported results are from the global model, which show that a 1-unit increase in social disadvantage is associated with a 0.279% increase in the rates of COVID-19 (*P* < .001). In contrast, the SESOI and convergence of vulnerability index had a negative relationship with rates of COVID-19 infection. The SDI has more spatial heterogeneity than the SESOI or the CVI (Figure 2). Rates of COVID-19 infections were localized to specific Miami–Dade census block groups and ranged from 0 to 60.75 per 1,000 population per square mile.

## Discussion

With the increasing number of COVID-19 cases in Miami–Dade County (from 62,430 cases on July 21, 2020, to 164,299 on September 15, 2020), a central focus of public health efforts should be limiting fatalities. In addition, exploring the heterogeneity of spatial relationships could provide more insights into place-based and policy-level strategies that protect vulnerable social groups. A limitation of this study is its reliance on the Florida Department of Health COVID-19 Dashboard; therefore, the reported cases may be an underestimation. Regardless, the methods used in this study demonstrate that geospatial analyses are powerful tools for estimating health events.
